# [Corrigendum] Ginkgolic acid inhibits the invasiveness of colon cancer cells through AMPK activation

**DOI:** 10.3892/ol.2025.15101

**Published:** 2025-05-21

**Authors:** Lina Qiao, Jianbao Zheng, Xianzhen Jin, Guangbing Wei, Guanghui Wang, Xuejun Sun, Xuqi Li

Oncol Lett 14: 5831–5838, 2017; DOI: 10.3892/ol.2017.6967

Subsequently to the publication of the above paper, an interested reader drew to the authors’ attention that certain of the western blot data in Fig. 4D on p. 5836 were strikingly similar to data that had previously appeared in the journal *Oncotarget* in a paper that featured some of the same authors. Moreover, a pair of the data panels showing the results of cellular migration and invasion assay experiments appeared to be overlapping between Figs. 2B and 4E, such that data which were intended to show the results from differently performed experiments had apparently been derived from the same original source.

However, the authors were able to consult their original data, and recognized how these errors occurred. Revised and corrected versions of Figs. 2 and 4, now showing replacement data for the scratch-wound assay and cell invasion assay experiments in Fig. 2A and B respectively, and new data for Fig. 4D and E, are shown on the next two pages. The authors regret the errors that were made during the compilation of the original figures, and are grateful to the editor of *Oncology Letters* for allowing them the opportunity to publish this Corrigendum. Note that the errors that were made in compiling this pair of figures did not have a significant impact on the conclusions reached in this study. All the authors agree with the publication of this corrigendum; furthermore, they apologize to the readership for any inconvenience caused.

## Figures and Tables

**Figure 2.**
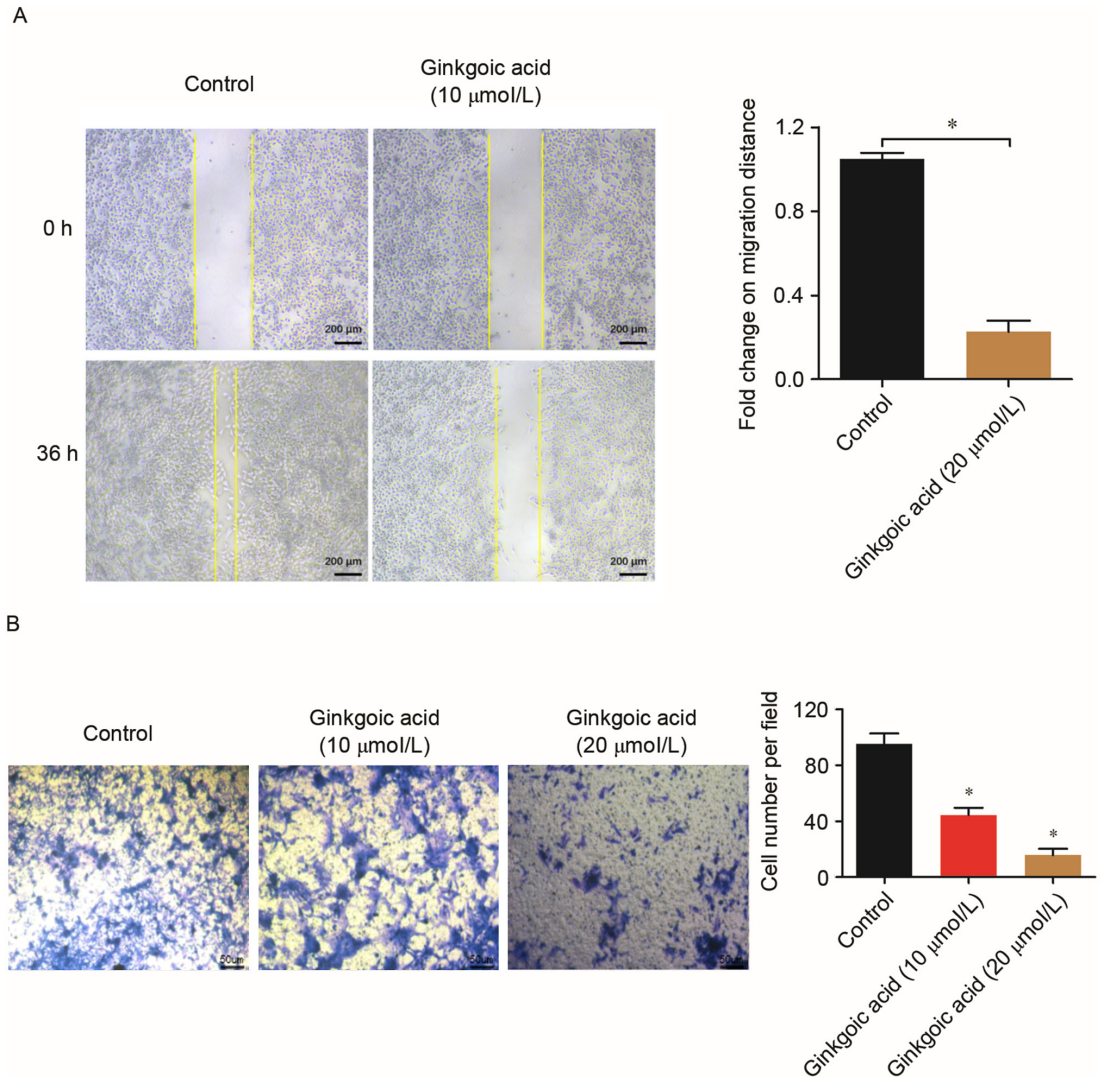
Effects of ginkgolic acid on the migration and invasion of SW480 cells. (A) SW480 cells at 90% confluence were treated with 10 µM ginkgolic acid or vehicle for 12 h, and a scratch assay was performed. Images were captured at 0 and 36 h at ×40 magnification. *P<0.05. (B) SW480 cells were seeded into a Matrigel-coated invasion chamber subsequent to treatment with ginkgolic acid at various concentrations (0, 10 and 20 µmol/l), and a Matrigel-invasion assay was performed at 48 h. The number of invaded cells was quantified by counting the cells from 10 random fields at ×20 magnification. Data are presented as the mean ± standard deviation. *P<0.05 vs. control.

**Figure 4.**
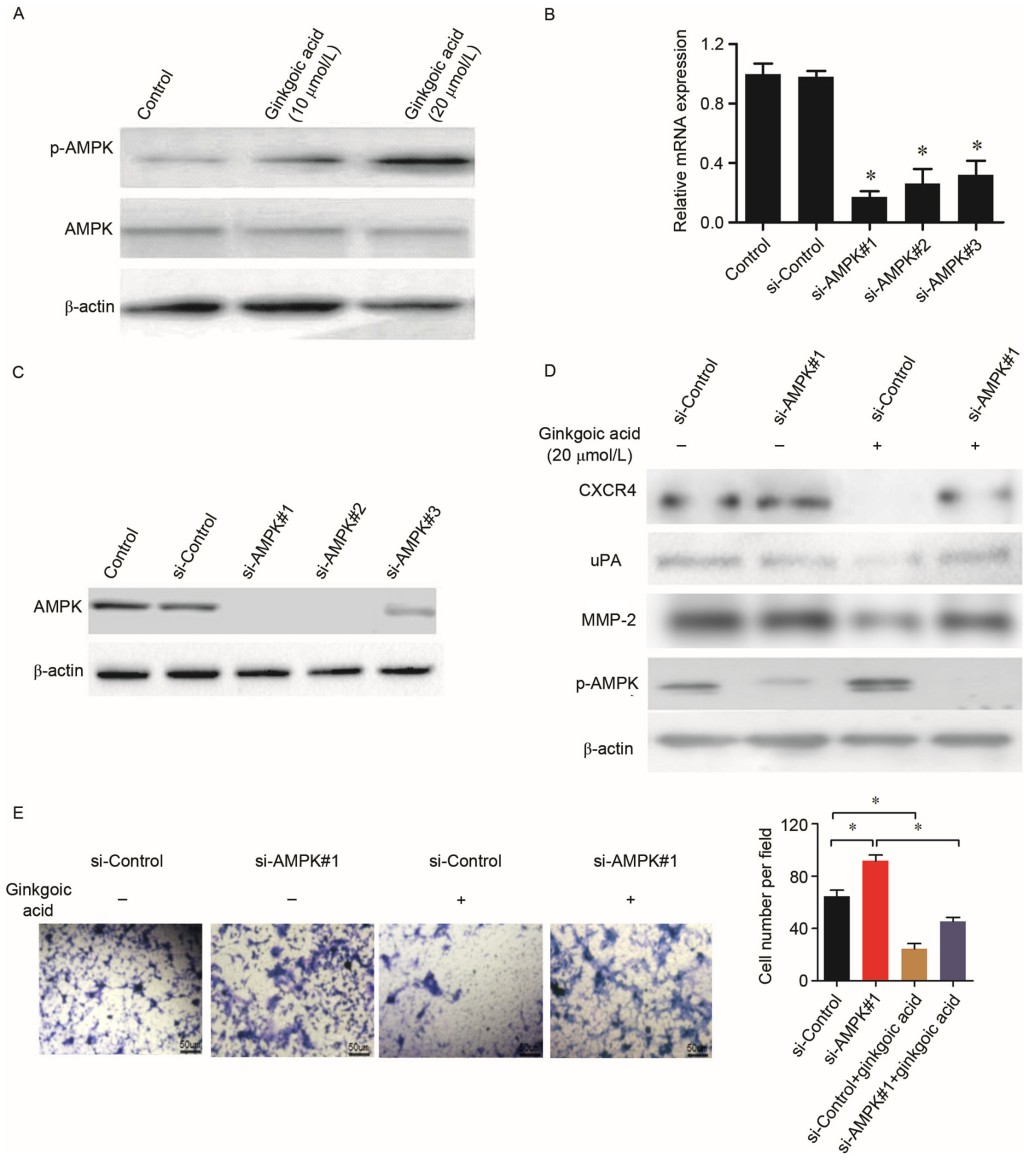
Ginkgolic acid-mediated downregulation of invasion-associated molecules in SW480 cells is associated with the activation of AMPK. (A) The expression of AMPK and p-AMPK in SW480 cells following ginkgolic acid treatment were detected by western blotting. siRNAs were used to silence AMPK expression in SW480 cells. The efficiency of siRNAs targeting AMPK was evaluated by (B) reverse transcription-quantitative polymerase chain reaction and (C) western blotting. (D) SW480 cells were treated with ginkgolic acid (20 µmol/l) for 48 h following AMPK silencing, and the expression of MMP-2, uPA, CXCR4 and p-AMPK were detected by western blotting. (E) The invasive ability of SW480 cells was detected by a matrigel-invasion assay following siRNA knockdown of AMPK with ginkgolic acid treatment. The number of invaded cells was quantified by counting the cells from 10 random fields at ×200 magnification. Data are presented as the mean ± standard deviation. *P<0.05. AMPK, adenosine 5’-monophosphate-activated protein kinase; p-, phosphorylated; siRNA, small interfering RNA; MMP, matrix metalloproteinase; uPA, urinary-type plasminogen activator; CXCR4, C-X-C chemokine receptor 4.

